# Akt and mTORC1 signaling as predictive biomarkers for the EGFR antibody nimotuzumab in glioblastoma

**DOI:** 10.1186/s40478-018-0583-4

**Published:** 2018-08-21

**Authors:** Michael W. Ronellenfitsch, Pia S. Zeiner, Michel Mittelbronn, Hans Urban, Torsten Pietsch, Dirk Reuter, Christian Senft, Joachim P. Steinbach, Manfred Westphal, Patrick N. Harter

**Affiliations:** 1 Dr. Senckenberg Institute of Neurooncology, University Hospital Frankfurt, Goethe University, Schleusenweg 2-16, 60528 Frankfurt am Main, Germany; 2 German Cancer Consortium (DKTK), Partner Site Frankfurt/Mainz, Frankfurt am Main, Germany; 3 German Cancer Research Center (DKFZ), Heidelberg, Germany; 4 Institute of Neurology (Edinger-Institute), University Hospital Frankfurt, Goethe University, Heinrich-Hoffmann-Str. 7, 60528 Frankfurt am Main, Germany; 5 Luxembourg Centre for Systems Biomedicine (LCSB), University of Luxembourg, Dudelange, Luxembourg; 6 Laboratoire national de santé (LNS), Dudelange, Luxembourg; 7 Luxembourg Centre of Neuropathology (LCNP), Dudelange, Luxembourg; 8 Department of Neuropathology, University of Bonn, Bonn, Germany; 9 Oncoscience GmbH, Schenefeld, Germany; 10 Department of Neurosurgery, University Hospital Frankfurt, Goethe University, Frankfurt am Main, Germany; 11 Department of Neurosurgery, University Hospital Hamburg Eppendorf, Martinistrasse 52, 20246 Hamburg, Germany

**Keywords:** Epidermal growth factor receptor, Mammalian target of rapamycin, Glioblastoma, Nimotuzumab, Biomarker, Targeted therapy

## Abstract

Glioblastoma (GB) is the most frequent primary brain tumor in adults with a dismal prognosis despite aggressive treatment including surgical resection, radiotherapy and chemotherapy with the alkylating agent temozolomide. Thus far, the successful implementation of the concept of targeted therapy where a drug targets a selective alteration in cancer cells was mainly limited to model diseases with identified genetic drivers. One of the most commonly altered oncogenic drivers of GB and therefore plausible therapeutic target is the epidermal growth factor receptor (EGFR). Trials targeting this signaling cascade, however, have been negative, including the phase III OSAG 101-BSA-05 trial. This highlights the need for further patient selection to identify subgroups of GB with true EGFR-dependency. In this retrospective analysis of treatment-naïve samples of the OSAG 101-BSA-05 trial cohort, we identify the EGFR signaling activity markers phosphorylated PRAS40 and phosphorylated ribosomal protein S6 as predictive markers for treatment efficacy of the EGFR-blocking antibody nimotuzumab in MGMT promoter unmethylated GBs. Considering the total trial population irrespective of MGMT status, a clear trend towards a survival benefit from nimotuzumab was already detectable when tumors had above median levels of phosphorylated ribosomal protein S6. These results could constitute a basis for further investigations of nimotuzumab or other EGFR- and downstream signaling inhibitors in selected patient cohorts using the reported criteria as candidate predictive biomarkers.

**Electronic supplementary material:**

The online version of this article (10.1186/s40478-018-0583-4) contains supplementary material, which is available to authorized users.

## Introduction

Glioblastoma (GB) is an incurable brain cancer and the most common primary brain tumor in adults [33]. The epidermal growth factor receptor (EGFR) is frequently genetically altered in GB by gene amplification and mutations including a variant where deletion of exons 2–7 causes activated signaling termed EGFR*vIII*. EGFR gene alterations can be found in 45.1% of GBs [32], mutations in members of the receptor tyrosine kinase- Ras-PI3 Kinase-AKT signaling network are the most frequent mutations (87.9% of cases) in GB [32]. Further, EGFR signaling is known to enhance proliferative signaling, resistance to cell death and reprogramming of energy metabolism [13, 38, 45]. Therefore, EGFR is a plausible target in GB therapy. Several clinical trials have been performed, with however rather disappointing results [39]. Strategies targeting EGFR in GB include small molecule inhibitors (e.g. erlotinib), antibodies or antibody-drug conjugates (e.g. depatuxizumab mafodotin (ABT-414)) as well as novel immunooncological approaches like a vaccine against EGFR*vIII* with rindopepimut. The depatuxizumab antibody portion of ABT-414 preferentially binds to cells with amplified EGFR or EGFR*vIII* [35]. After binding ABT-414 is internalized and can block microtubule formation via its mafodotin part [51]. Currently larger phase II and III clinical trials are underway evaluating ABT-414 in the primary (Intellance 1 phase III trial, ClinicalTrials.gov NCT02573324) and recurrent disease (Intellance 2 phase II trial, ClinicalTrials.gov NCT02343406) setting. In the ACT IV trial, the EGFR*vIII* vaccine rindopepimut did not prolong survival in GB patients [53]. It is noteworthy that the EGFR*vIII* mutation if present usually is only found in a fraction of tumor cells within a GB [54] and that even during the course of standard treatment EGFR*vIII* is frequently lost [53]. Standard treatment for patients in sufficient clinical condition has been established in 2005 already and involves surgical resection, radiotherapy and chemotherapy with the alkylating agent temozolomide which led to median overall survival times of 14.6 months [47]. Many trials have been conducted in recent years, however, no new drugs have been approved [27, 39]. Histologically, GB is characterized by marked hypoxic areas, with typical histological features of neoangiogenesis and necrosis in a diffusely infiltrating growing glial tumor [25]. These areas reflect the metabolically challenging microenvironment where nutrient and oxygen supply can frequently not match demand of the tumor cells. The transcription factor hypoxia-inducible factor 1α (HIF-1α) is a major cellular regulator of adaptive programs to hypoxia and stabilization occurs when oxygen is low [42].

The current WHO classification further stratifies GB as either isocitrate dehydrohgenase (IDH) wildtype (wt) or IDH mutant (mut). The vast majority of primary GB harbors IDH wt status [24]. Further, current treatment relevant molecular stratification of GB mainly depends on the methylation status of the O(6)-methylguanine methyltransferase (MGMT)-promoter. MGMT-promoter methylation correlates with reduced expression of the DNA repair enzyme MGMT. Consequently, tumors with methylated MGMT promoter generally respond better to temozolomide treatment whereas MGMT expression in tumors with unmethylated gene promoter is a major mechanism of resistance and indicator for poor prognosis [15, 16, 46].

**Fig. 1:**
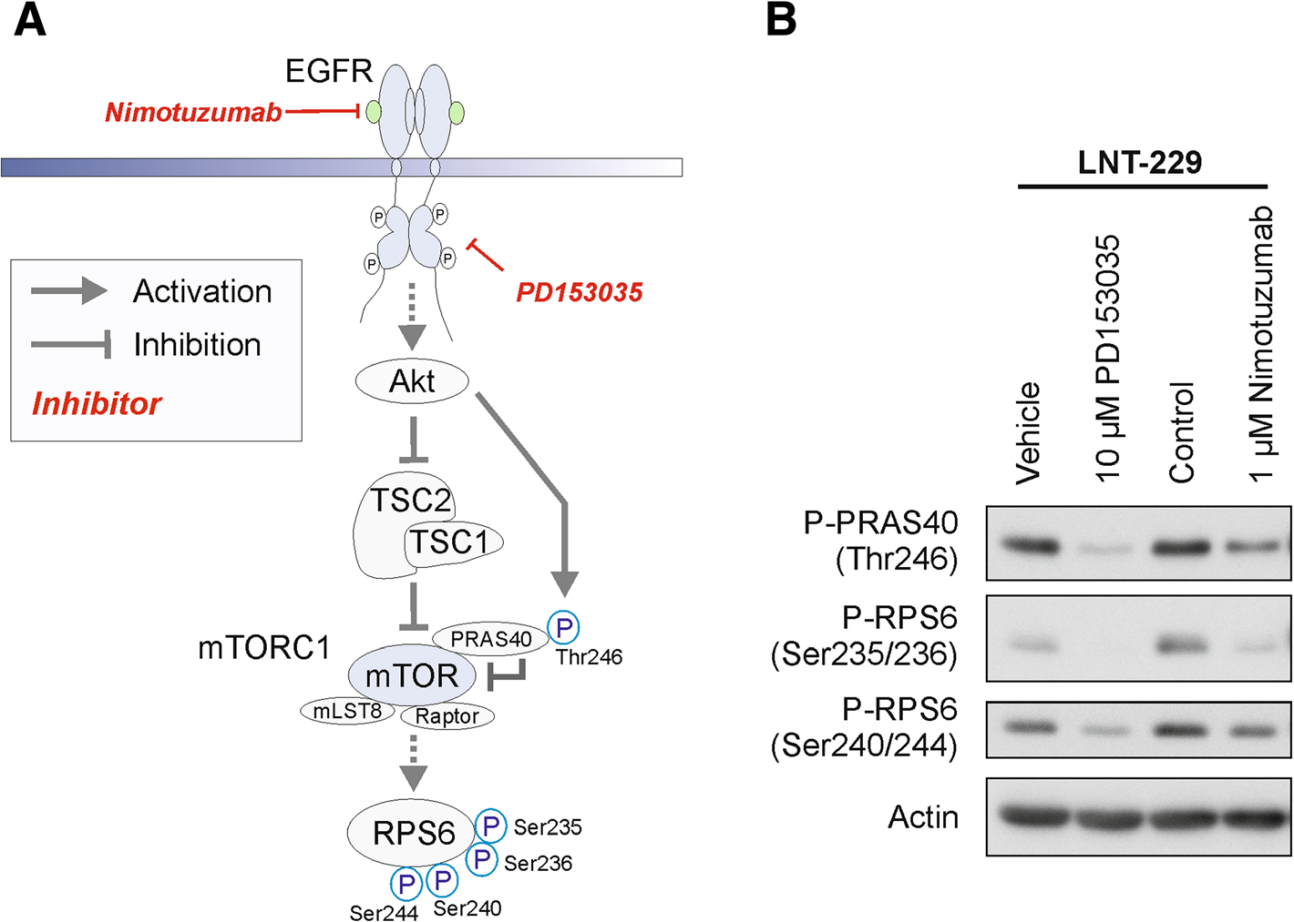
EGFR signal transduction and effects of EGFR inhibition on downstream targets. **a** Scheme of EGFR signal transduction. Nimotuzumab and PD153035 are inhibitors of EGFR: Activation of EGFR results in activation of Akt signaling which relieves a TSC1/TSC2 as well as PRAS40 (via phosphorylation of Thr246) -mediated inhibition of mTORC1. RPS6 phosphorylation at Ser235/236 and Ser 240/244 is regulated by mTORC1. **b** LNT-229 cells were incubated in serum-free DMEM for 90 min with vehicle (DMSO control), PD153035 (dissolved in DMSO), control solution for nimotuzumab (placebo solution of the trial) or 1 μM nimotuzumab as indicated. Cellular lysates were analyzed by immunoblot with antibodies as indicated

Many novel approaches to improve GB therapy rely on targeting specifically altered signal transduction cascades. However, these so called targeted therapies, including those targeting EGFR, thus far, have failed to show any benefit in GB treatment despite rational target selection and availability of potent drugs opening the quest for predictive biomarkers [39, 52]. One important downstream mediator of EGFR signaling is the kinase Akt (Fig. 1a) with numerous phosphorylation targets involved in proliferation, survival, cell motility and angiogenesis [49]. Proline rich Akt substrate of 40 kDa (PRAS40) has been identified as an inhibitory component of mTOR complex 1 (mTORC1). Akt is the main regulator of phosphorylation at Thr246 and relieves PRAS40-mediated inhibition of mTORC1 (Fig. 1a) [23, 41]. PRAS40-phosphorylation correlated with shorter time to progression in a smaller GB patient cohort [8]. Another study in low grade glioma found a trend towards shorter survival in tumors with higher phospho-PRAS40 levels; however, statistical significance was not reached [29]. Besides its regulation via PRAS40 phosphorylation, Akt also activates mTORC1 via inhibitory phosphorylation of a protein complex consisting of tuberin (TSC1), hamartin (TSC2) as well as the more recently discovered TBC1D7 (this complex will be termed in TSC1/2 in the following text for simplicity reasons) (Fig. 1a) [10, 19]. MTORC1 additionally integrates signals from the cellular energy status including oxygen availability [4], amino acid availability [2] and direct ATP content of the cell [20]. The ribosomal protein S6 (RPS6) is a downstream effector of mTORC1 and is part of the ribosomal machinery. RPS6 phosphorylation has been discovered many years ago, still its molecular and physiological effects especially with regard to the phosphorylation of the different serine sites are currently still under investigation [31]. RPS6 has several mTORC1-dependent phosphorylation sites including serines at position 235 and 236 as well as the highly specific position 240 and 244 (Fig. 1a) [31, 34].

Nimotuzumab is a blocking monoclonal antibody against EGFR [48] without intrinsic EGFR activating activity. It has shown promising results as a targeted therapy in the treatment of high grade gliomas in phase II studies [3] and pediatric brain stem gliomas [28, 57]. Therefore, a two arm phase III clinical trial (OSAG 101-BSA-05) involving 149 patients was performed comparing standard (radiotherapy and temozolomide) treatment with and without addition of nimotuzumab (EudraCT No. 2005–003101-85, ClinicalTrials.gov NCT00753246) [55]. Nimotuzumab was administered once weekly (400 mg) during the concomitant radio-temozolomide phase and afterwards continued biweekly (400 mg) for 12 weeks during the adjuvant temozolomide treatment phase. The trial was negative, and a benefit of nimotuzumab treatment was apparent neither in the whole population studied nor in patients with EGFR amplification. A post-hoc analysis of subgroups, however, revealed a trend for improved survival for MGMT unmethylated patients with residual tumor when treated with nimotuzumab (PFS 6.2 vs. 4.0 months; OS 19.0 vs. 13.8 months). This unplanned subgroup analysis, however, included only 28 patients and failed to reach statistical significance. The results of several recent trials suggest that for an effective targeted therapy, appropriate patients need to be identified [56]. With regard to signal transduction inhibitors it is plausible that genetic heterogeneity in GBs is also reflected by different degrees of dependence on certain signaling cascades [32]. The aim of this study was to analyze EGFR-dependent Akt and mTORC1 signaling in treatment-naïve tumor samples of the OSAG 101-BSA-05 patient cohort as a potential predictive biomarker of nimotuzumab efficacy. We analyzed the response to nimotuzumab therapy of molecular subgroups depending on activation of Akt and mTORC1 signaling, extent of necrosis, HIF-1α staining and MGMT-methylation status. We here report a predictive signature of RPS6 and PRAS40 phosphorylation in MGMT unmethylated patients. Furthermore, we describe a trend for a predictive value of RPS6 phosphorylation in all patients irrespective of MGMT promoter methylation status.

## Materials and methods

### Reagents

Nimotuzumab as well as the corresponding placebo control solution were provided by Oncoscience (Wedel, Germany). Nimotuzumab is an IgG subtype 1 kappa with a molecular weight of 147.613 kDa. The EGFR inhibitor PD153035 [11] was purchased from Sigma Aldrich (Taufkirchen, Germany).

### Cell culture

LNT-229 GB cells have been described previously [38, 50] and were maintained in Dulbecco’s modified eagle medium (DMEM) containing 10% foetal calf serum (FCS) (Biochrom KG, Berlin, Germany), 100 IU/ml penicillin and 100 mg/ml streptomycin (Life Technologies, Darmstadt, Germany).

### Immunoblot

Immunoblot was performed as described previously [14]. 10 μg of protein per condition were used for SDS-PAGE analysis. Membranes were incubated with antibodies against phospho-RPS6 (Ser 240/244) (D68F8; Cell Signaling), phospho-RPS6 (Ser 235/236) (D57.2.2.E; Cell Signaling), phospho-PRAS40 (Thr246) (C77D7, Cell signaling) or actin (# sc-1616 Santa Cruz Biotechnology, Dallas, Texas, USA). The secondary HRP-conjugated antibodies were purchased from Santa Cruz Biotechnology (Dallas, Texas, USA). A chemiluminescence solution was used for detection [50].

### Patients, sample collection and immunohistochemistry

The OSAG 101-BSA-05 study (EudraCT No. is 2005–003101-85, ClinicalTrials.gov

NCT00753246) cohort included 149 patients with GB [55]. This open label, randomised phase III study was approved by the central and local ethics review boards. Informed consent was obtained from all patients. In case of availability, we obtained tissue sections from these tumors for further immunohistochemistry. We investigated the amount of necrosis (%) in hematoxylin and eosin (HE)-stained slides of the tissue sections (*n* = 111), HIF-1α expression (%) in the vital tumor centre (*n* = 106) as well as in perinecrotic areas (*n* = 98), P-PRAS40-positive cells (%) (*n* = 101), P-RPS6-positive cells (%) (*n* = 109) as well as Iba1-positive cells (%) (*n* = 100) using standard procedures on an automated IHC staining system. Stainings with antibodies against threonine 246-phosphorylated PRAS40 (P-PRAS40) and serine 240/244-phosphorylated RPS6 (P-RPS6) (Cell signaling, #2997 and #5364 respectively) were performed as recently reported [14]. Furthermore, the following antibodies were used: HIF-1α (Novus Biologicals, NB 100–134), Iba1 (Wako, 019–19,741). Samples that consisted of 100% necrosis were excluded from further analysis.

### Statistical analyses

Statistical analyses were performed using JMP version 13 software (SAS Institute, Heidelberg, Germany). A *p*-value of *p* < 0.05 was chosen to declare statistical significance. Applied statistical test methods are either mentioned in the figure legend or in the flow content. For dichotomized univariate survival analyses we performed a median split to obtain a high and low group with regard to the investigated factor. The high group includes specimen with values above median, the low group includes specimen with median or below.

## Results

### Nimotuzumab inhibits EGFR downstream signaling

To test whether nimotuzumab inhibited signaling from the EGFR-downstream kinases Akt and mTORC1 (Fig. 1a), we exposed human LNT-229 glioblastoma cells to nimotuzumab or the intracellular EGFR inhibitor PD153035 [11]. Both substances caused effective inhibition of EGFR downstream signal transduction indicated by a similar degree of reduction in phosphorylation of the corresponding target proteins PRAS40 as well as RPS6 in an immunoblot experiment (Fig. 1b). We chose P-PRAS40 (Thr246) and P-RPS6 (Ser240/244) in our further tissue analysis due to the specificity of the phosphorylation site and the availability of robust, monoclonal antibodies for IHC. Effective Akt inhibition by nimotuzumab had also previously been reported in other cell lines including EGFR overexpressing U87 GB cells, lung and nasopharyngeal carcinoma cells [7, 18, 37].

### Phosphorylation of PRAS40 and RPS6 is only detectable in a small proportion of tumor cells and does not correlate with EGFR gene amplification

**Fig. 2:**
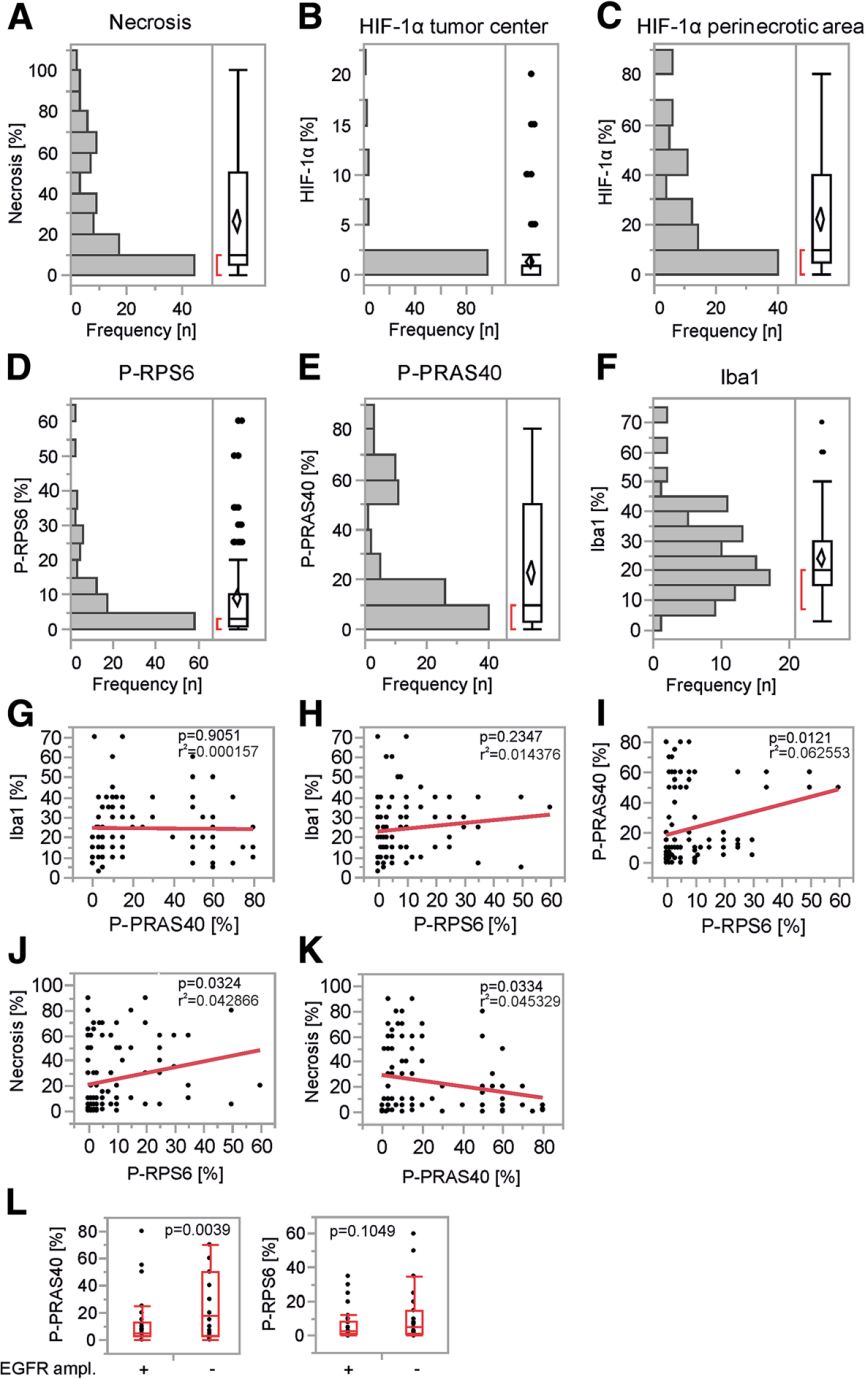
Histological characterization of the patient cohort. **a**-**f**, outlier box plot for the distribution of necrosis, HIF-1α in vital, central or perinecrotic tumor areas, phosphorylated (P-)RPS6, P-PRAS40 and Iba1 in samples as indicated (horizontal line within the box is the median sample value; confidence diamond contains the mean and the upper and lower 95% of the mean; ends of the box represent the 25th and 75th quantiles; bracket outside of the box is the shortest half, which is the most dense 50% of observations). **g**-**k**, correlations of histological markers as indicated in a bivariate plot with a linear regression analysis. P and r^2^ values as indicated. **l** one way analysis with outlier box plot of P-PRAS40 and P-RPS6 in EGFR amplified vs. non-amplified tumor specimens. *P*-value calculated using Student’s *t*-test

For histological characterization of our cohort, we evaluated the extent of necrosis, P-PRAS40, P-RPS6 and HIF-1α in perinecrotic as well as in vital tumor areas. Additionally, we analyzed Iba1 expression as a marker for glioma-associated microglia and macrophages (GAMs) and potential source of P-PRAS40 and P-RPS6 expression (Additional file 1: Figure S1). Extent of necrosis ranged from 0 to 100%, with a median of 10% (Fig. 2a). HIF-1α within central vital tumor areas was undetectable in most tumors but ranged up to 20% in one tumor with a median of 0% (Fig. 2b). In contrast, perinecrotic HIF-1α ranged from 0 to 80% with a median of 10% (Fig. 2c) and correlated with necrosis extent (Additional file 2: Figure S2). P-PRAS40 was detectable in a fraction of cells with a range of 0 to 80% and a median of 10% (Fig. 2d). P-RPS6 was similarly detectable in a fraction of tumor cells with a similar range of 0 to 60% however the median was lower at 3% (Fig. 2e). Besides the actual GB tumor cells, GAMs can account for a relevant fraction of intratumoral cells and potentially influence signal transduction of cancer cells or constitute a potential source of mTORC1 or AKT signaling. Therefore, we stained the samples for the pan-microglia and macrophage (M/M) marker Iba1. Staining frequency ranged from 3 to 70% with a median of 20% (Fig. 2f). Neither P-PRAS40 nor P-RPS6 correlated with Iba1 (Fig. 2g, h). In contrast, P-PRAS40 and P-RPS6 expression as markers of EGFR signal transduction correlated (Fig. 2i). Besides being downstream of EGFR, mTORC1 is also regulated by the cellular energy charge and nutrient supply [20, 40]. GB necrosis occurs where demand exceeds supply of the fast growing tumor cells and the perinecrotic area is where nutrient and oxygen deprivation are most severe within the tumor. Interestingly, P-RPS6 as a target of mTORC1 was increased in necrotic tumors potentially indicating a defective nutrient sensing as a cause of increased necrosis [50] (Fig. 2j). An inverse correlation was found for P-PRAS40 (Fig. 2k). Neither P-PRAS40 nor P-RPS6 correlated with Hif-1α staining (data not shown).

Information on EGFR amplification and vIII mutation was available for 88 and 81 cases respectively [55]. EGFR gene amplification correlates with increased expression of EGFR [43] and was found in 43 cases. An inverse effect was detectable on downstream Akt but not mTORC1 signal transduction (Fig. 2l). However, with only 7 cases of vIII mutation in our cohort, the number was too small to derive any conclusions in this regard. Notably, there was also no difference in the end points for patients with and without EGFR amplification or vIII mutation in the OSAG 101-BSA-05 trial [55].

### Necrosis extent and HIF-1α staining is not associated with patient survival

While necrosis as a surrogate of hypoxia or ischemia is a common histological feature in GB, a more outspread or increased necrosis extent or hypoxia could indicate a particularly aggressive tumor subtype. A relationship between patient survival and intratumoral hypoxia has e.g. been reported for uterine cancer [17]. In our cohort, we did not find an association between necrosis extent or HIF-1α staining and patient survival in univariate Weibull parametric survival analysis (Table 1). EGFR signaling is known to promote many components of a more aggressive tumor phenotype and P-PRAS40 has been reported as an independent prognostic marker with regard to time to progression in a small glioma cohort [8]. Neither P-RPS6 nor P-PRAS40 staining correlated with overall survival (Table 1).

**Table 1:** Correlation of histology markers with survival

Treatment arm	Parametric survival Weibull p					
	Necrosis	HIF-1α perinecrotic area	HIF-1α vital tumor	P-PRAS40	P-RPS6	Iba1
Nimotuzumab	0.7360	0.3733	0.6135	0.2365	0.6078	0.5149
Control	0.1003	0.4436	0.7257	0.6929	0.2967	0.0275

Univariate Weibull parametric survival analysis was performed for the listed parameters

### Treatment of hypoxic tumors with nimotuzumab is not detrimental

**Fig. 3:**
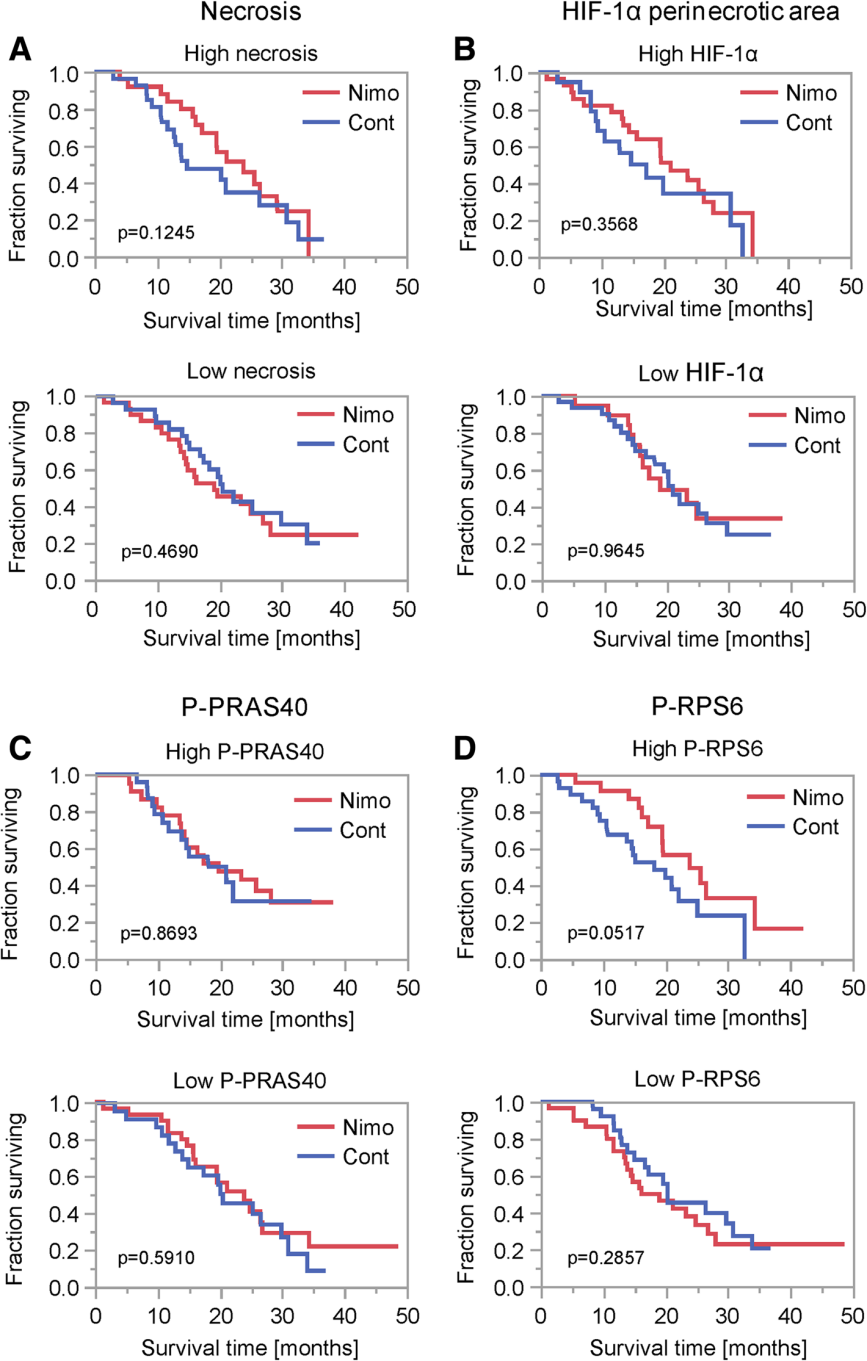
Survival analyses depending on treatment in histological subgroups. **a**-**d** Kaplan-Meier survival curves for patients treated with nimotuzumab (nimo) or placebo (cont) in dichotomized histological subgroups (median split, above median: high, below and equal to median low) for necrosis (**a**), HIF-1α in perinecrotic regions (**b**), P-PRAS40 (**c**) and P-RPS6 (**d**). *P* values were calculated using the Wilcoxon test

We have previously shown that inhibition of EGFR or mTORC1 signal transduction can protect human glioblastoma cells from hypoxia-induced cell death [38, 45]. Therefore, we hypothesized that in tumors with increased necrosis or HIF-1α staining, nimotuzumab could mediate tumor-protective effects resulting in decreased survival of patients. Necrosis extent, HIF-1α staining, P-PRAS40, P-RPS6 and Iba1 staining were well-balanced between the two treatment arms (Additional file 3: Figure S3A). Using a median split, we dichotomized tumors into two groups (high and low) (Additional file 1: Figure S1). Within the group of above median value necrotic tumors, nimotuzumab treatment resulted in a slight trend towards improved survival, whereas no trend was detectable in below or median value necrotic tumors (Fig. 3a). Also, no trend was detectable with regard to HIF-1α high and low tumors (Fig. 3b). Even though P-PRAS40 and P-RPS6 were not associated with patient survival in the treatment arms (Table 1), tumors with activated downstream signaling might define a patient subgroup more addicted to EGFR signaling and thus more prone to respond to nimotuzumab. There was no trend in overall survival in tumors with high or low P-PRAS40 with regard to nimotuzumab therapy (Fig. 3c). In contrast in P-RPS6 high tumors, we observed a clear trend towards improved survival when nimotuzumab treatment was administered (Fig. 3d).

### Unmethylated MGMT promoter status defines a subgroup in which high necrosis, P-RPS6 or P-PRAS40 tumors benefit from nimotuzumab treatment

**Fig. 4:**
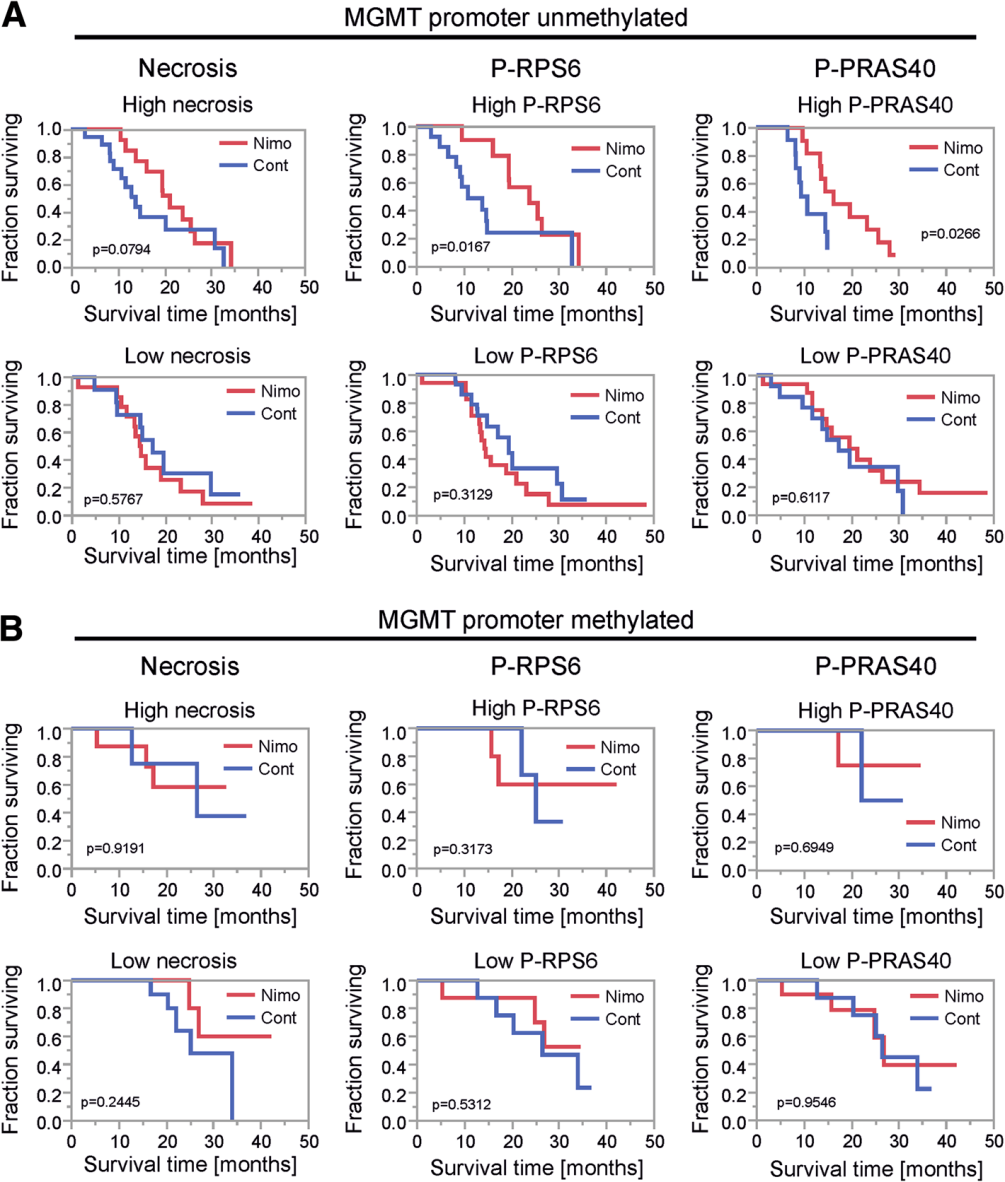
Survival analyses depending on treatment in histological subgroups for the MGMT-promoter unmethylated and methylated tumor cohort. **a**-**b** Kaplan-Meier survival curves for patients treated with nimotuzumab (nimo) or placebo (cont) in dichotomized histological subgroups (median split, above median: high, below and equal to median low) for necrosis, P-PRAS40 and P-RPS6 in the MGMT-promoter unmethylated (**a**) and methylated (**b**) tumor cohort. P values were calculated using the Wilcoxon test

In accordance with previous results, MGMT promoter methylation status was associated with patient survival in the OSAG 101-BSA-05 study cohort [55]. To test if the difference in biological behavior was also reflected by different activities of Akt and mTORC1 signaling, we investigated P-PRAS40 and P-RPS6 in both tumor subgroups. There was no difference in staining frequency for P-PRAS40 and P-RPS6 in MGMT promoter methylated vs. unmethylated tumors (Additional file 3: Figure S3B). In MGMT unmethylated GBs a treatment effect might be to a lesser extent concealed by temozolomide efficacy. When considering only the MGMT unmethylated cohort, the clear trend in favor of nimotuzumab therapy already detectable in the whole cohort regardless of MGMT promoter methylation status, now became significant when using a median split for P-RPS6 in tumors with above median value (*p* value of 0.02, Wilcoxon) (Fig. 4a). Additionally, the same effect was also detectable when using a P-PRAS40 median split in the MGMT promoter unmethylated tumor cohort (*p* = 0.03, Wilcoxon) (Fig. 4a). Also, there was a trend towards an efficacy of nimotuzumab in MGMT promoter unmethylated tumors with above median extent of necrosis (Fig. 4a). No effect was detectable in tumors with below or median values for necrosis, P-RPS6 and P-PRAS40 (Fig. 4b).

### P-RPS6 expression predicts survival depending on the treatment group in MGMT promoter unmethylated GBs

**Fig. 5:**
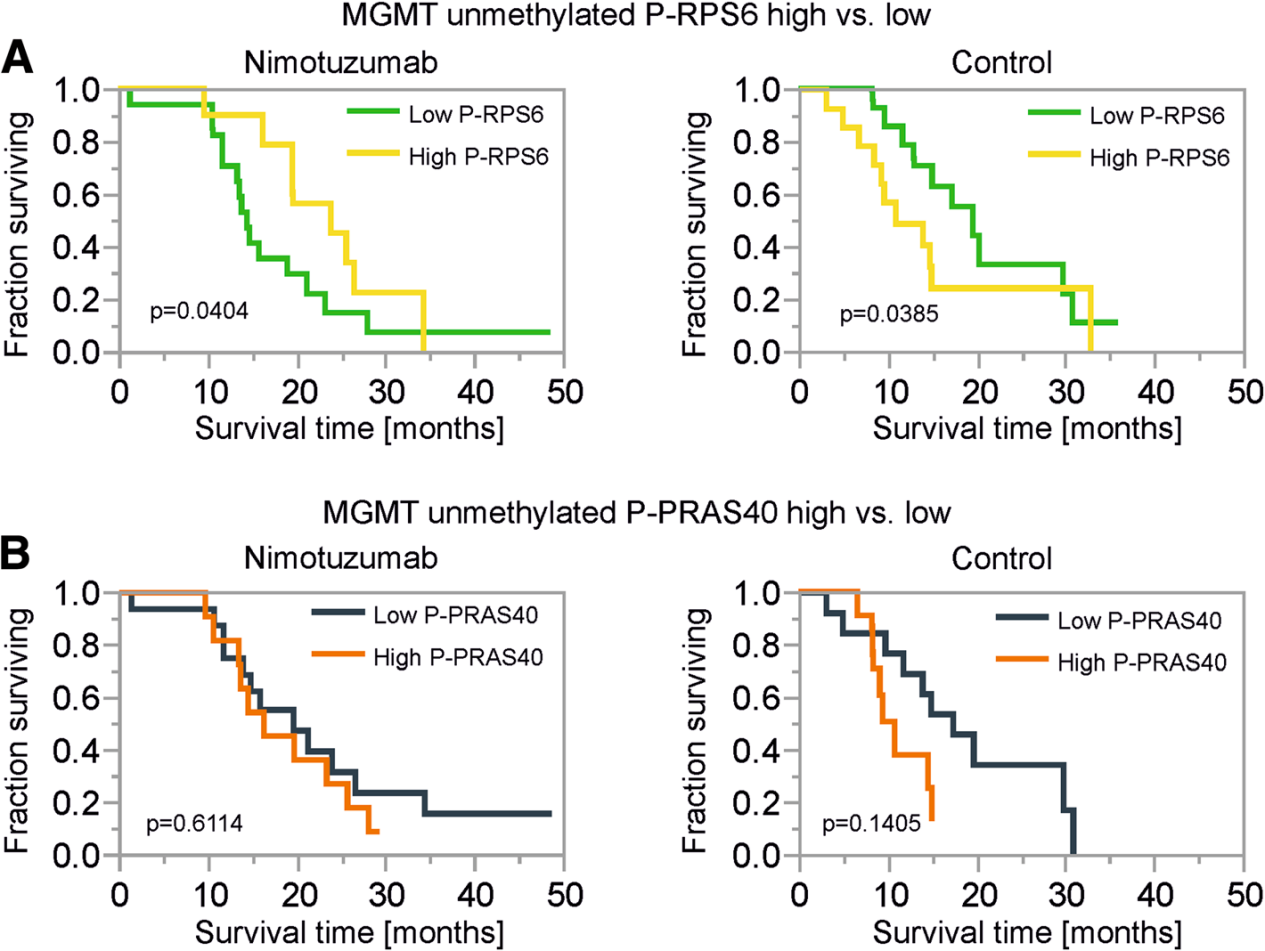
Prognostic relevance of P-RPS6 and P-PRAS40 in treatment groups of MGMT-promoter unmethylated tumors. **a**-**b** Kaplan-Meier survival curves for patients with MGMT promoter unmethylated GBs treated with nimotuzumab or placebo (control) for dichotomized histological subgroups (median split, above median: high, below and equal to median low) for P-RPS6 (**a**) and P-PRAS40 (**b**). P values were calculated using the Wilcoxon test

We wondered whether P-RPS6 was also relevant for survival of patients within the treatment arms in MGMT promoter unmethylated GBs. In patients treated with nimotuzumab, an above median expression of P-RPS6 was associated with improved survival (Fig. 5a, left panel). In contrast in patients with control treatment, above median P-RPS6 expression was associated with reduced survival (Fig. 5a, right panel). No association of P-PRAS40 with patient survival within the treatment arms was detectable when using a median split (Fig. 5b).

### Increased GAM levels correlate with improved survival in patients treated with nimotuzumab

**Fig. 6:**
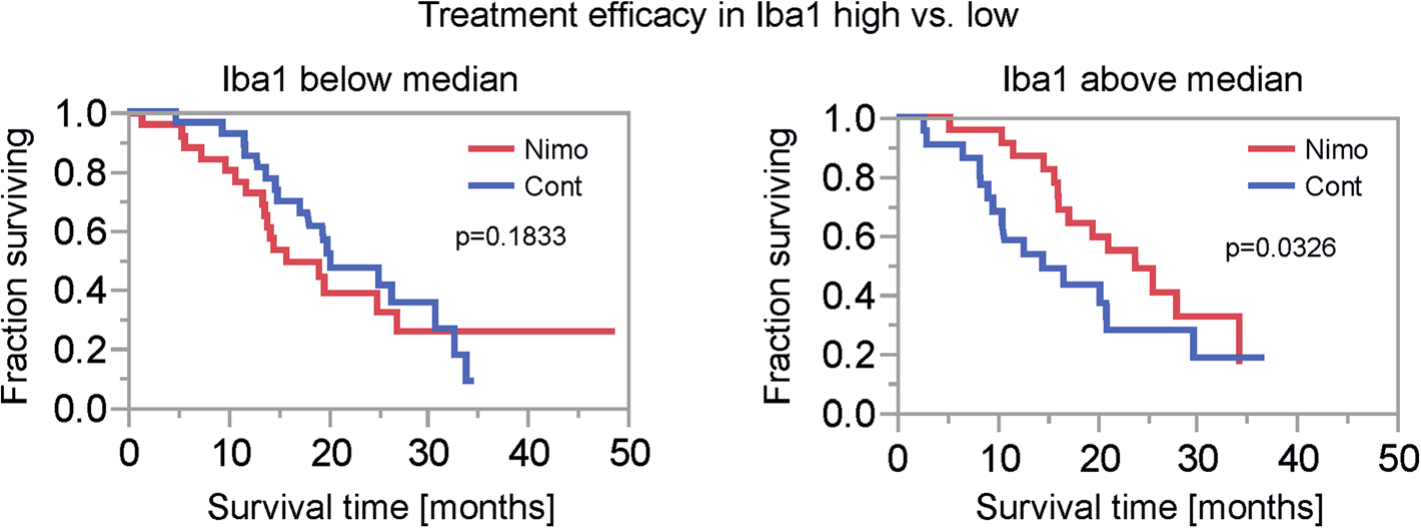
Survival analysis depending on treatment in subgroups based on microglial prevalence. Kaplan-Meier survival curves for patients treated with nimotuzumab (nimo) or placebo (cont) in dichotomized subgroups based on Iba1 staining frequency (median split, above median: high, below and equal to median low). P values were calculated using the Wilcoxon test

GAMs constitute relevant portions of a GB and the assumption that GAMs might be associated with an adverse prognosis in GB patients is under debate [44]. Interestingly, in tumors with above median Iba1 staining frequency (Iba1 high), nimotuzumab treatment was associated with a prolonged survival (Fig. 6, right panel). In contrast no effect of nimotuzumab was detectable for tumors with below median Iba1 staining frequency (Iba1 low) (Fig. 6, left panel).

## Discussion

The experience with targeted therapies in recent GB trials has been overall disappointing highlighting the need for predictive biomarkers. In this retrospective analysis of samples of the OSAG 101-BSA-05 trial [55], we investigated histological subgroups based on necrosis and hypoxia as markers for a nutrient-deprived tumor microenvironment as well as for phosphorylation of PRAS40 and RPS6 as downstream markers of EGFR signaling. We hypothesized a reduced efficacy of EGFR inhibition therapy in tumors with pronounced necrosis or hypoxia due to potential protective effects of inhibitor therapy in this context [38, 45]. Tumor hypoxia as indicated by HIF-1α staining as well as necrosis were not associated with patient survival (Table 1). When using a median split for necrosis extent, on the contrary to our hypothesis, there was a slight trend towards improved efficacy of nimotuzumab in patients with tumors with above median necrosis (Fig. 3a). No trend was detectable using a median split for perinecrotic HIF-1α staining frequency (Fig. 3b). HIF-1α staining frequency in vital tumor tissue was low with a median of 0%, therefore we did not include a dichotomized analysis in our study. GBs with increased signaling from EGFR and downstream kinases might constitute a collective with oncogene addiction exposing an Achilles heel for targeted therapies. Dichotomizing for P-PRAS40 high and low tumors had no effect on nimotuzumab treatment efficacy (Fig. 3c), in contrast to P-RPS6 where a clear trend towards nimotuzumab efficacy was detectable in tumors with high P-RPS6 (Fig. 3d). Neither P-PRAS40 nor P-RPS6 was associated with patient survival (Table 1). However, when testing for time to progression, P-PRAS40 was associated with a shorter interval (Additional file 4: Figure S4A) similar to a previous report [8].

The majority of GB (approximately 55–65%) has an unmethylated MGMT promoter defining a subgroup that is especially difficult to treat due to the reduced efficacy of temozolomide [9, 15, 22]. When investigating only MGMT unmethylated tumors, above median P-RPS6 was associated with nimotuzumab efficacy (Fig. 4a) which has already been detectable as a trend in the whole study cohort (Fig. 3a, d). In addition, above median P-PRAS40 was associated with improved survival in patients treated with nimotuzumab (Fig. 4a). The positive correlation between necrosis extent and P-RPS6 (Fig. 2j) was unexpected considering that mTORC1 is also a component of central cellular nutrient sensing pathways and cells with intact nutrient sensing inhibit mTORC1 in nutrient deplete conditions [50]. This indicates a potentially dysregulated mTORC1 sensor in our cohort resulting in higher extent necrosis as has been suggested recently (Additional file 4: Figure S4B) [50]. The efficacy of nimotuzumab in patients with high P-RPS6 (as a trend in the whole study cohort and statistically significant only in MGMT unmethylated GBs) points to a potentially higher degree of addiction to mTORC1 and ultimately EGFR signaling in this subgroup. While the homogeneous patient cohort of a registered randomized phase III trial adhering to central monitoring standards was a major strength of our study, introducing subgroups naturally shrunk patient numbers and our results need to be validated prospectively in a larger patient cohort using our EGFR signaling markers as entry criteria. Additionally, PTEN and PI3 Kinase loss/mutation are frequent events in GB (~ 36% and ~ 6% of GB samples respectively) [32] and most likely partly impact nimotuzumab efficacy. Therefore, it is remarkable that P-RPS6 dichotomization was sufficient to define a subgroup with a clear trend towards nimotuzumab efficacy in samples of unknown PTEN and PI3 Kinase status (Fig. 3). In an upcoming prospective analysis, it would be important to include these markers and PTEN and PI3 Kinase wildtype status would most likely define an even more nimotuzumab-susceptible subgroup of tumors. Accordingly, in a previously published retrospective analysis of tissue of 26 GB patients treated with the non-antibody EGFR inhibitors erlotinib or gefitinib response in the recurrent disease setting correlated with expression of vIII-mutated EGFR and PTEN [30]. No evaluation of downstream phosphorylation events in the tumor tissue was included in this analysis, still these results suggest that tumors with high EGFR signaling activity and intact signal transduction are sensitive to EGFR inhibitors. In the recent phase II EORTC 26082 trial, similar to our results, mTORC1 activation as indicated by phosphorylation of the mTOR protein itself at Ser2448 was a marker to predict response to treatment with the mTOR inhibitor temsirolimus in MGMT unmethylated GBs [56]. The relevant kinase that mediates phosphorylation of mTOR at Ser2448 is S6 Kinase [5] which is exactly the same kinase that mediates RPS6 phosphorylation and therefore is responsible for P-RPS6 in our cohort (Additional file 4: Figure S4C). Additionally, in multivariate analyses, PRAS40 phosphorylation was associated with survival in the temsirolimus treatment arm [56]. The authors propose phosphorylated mTOR (Ser2448) and P-PRAS40 as potential biomarkers for mTOR inhibitor therapy in MGMT-promoter unmethylated GBs. Our results confirm this notion with nimotuzumab as an indirect mTORC1 inhibitor (Fig. 1b). Integrating the results of the analyses of predictive signatures for EGFR [30] and mTOR inhibitors [56] and our analysis points to a signature where a high (er) degree of activation and an intact EGFR signaling axis defines GBs susceptible to inhibitors of this pathway in general. Accurate analysis of the in vivo phosphorylation status of proteins by IHC to monitor EGFR signaling activity requires special caution. E.g. time to processing and several other factors can have a major influence on phosphorylation and dephosphorylation events [14]. Therefore, for a prospective analysis of biomarkers in a clinical trial, standardized tissue asservation will be an important topic to include in the protocol.

The need for and potential adverse effects of neglecting potential predictive biomarkers is highlighted by the recently published results of the thus far largest randomized phase II trial evaluating the efficacy of the mTORC1 inhibitor everolimus in newly diagnosed GB that randomized 171 patients [6]. Patients receiving everolimus in addition to standard radiochemotherapy in this trial had a reduced survival in comparison to sole standard radiochemotherapy [1, 6]. One potential explanation of these results demonstrating reduced survival when an mTOR inhibitor was added to the therapeutic regimen in GB could be protective effects of mTOR inhibition in the context of the tumor microenvironment that we have previously shown in cell culture models [38].

Data regarding the prognostic impact of the innate immune system including GAMs in GBs is conflicting [12, 21]. In our study cohort, we found a positive effect on prolonged overall survival in patients treated with nimotuzumab with GBs of above median Iba1 frequency (Fig. 6). Investigating the whole patient cohort irrespective of treatment arm, we found no association with survival when dichotomizing for high vs. low GAM infiltration (Additional file 4: Figure S4D). These results contrast the notion that GAM subpopulations might have negative effects on GB patient survival [36]. However, similar findings as in our cohort regarding the prognostic role of GAMs are described, likewise demonstrating a positive prognostic impact of at least a GAM subpopulation in GB [58]. Currently we can only speculate on the underlying reasons for this positive effect of intratumoral GAMs on overall survival in GB patients treated with nimotuzumab. It is interesting to note that microglia express receptors for binding of the Fc part of antibodies and might therefore react with nimotuzumab-bound GB cells similar to mechanistic hypotheses of antibody mediated plaque clearance in Alzheimer’s models [26]. Further clarifying potential antibody effects on GAMs is beyond the scope of this article and should be investigated elsewhere.

## Conclusions

The quest for new treatment options in GB has been cumbersome at best with no new drugs gaining approval since the introduction of temozolomide. In this current study, we investigated tissue samples of yet another negative phase III trial. The EGFR is one of the most plausible treatment targets in this cancer entity. We here report markers for the selection of patients that might benefit from the EGFR-blocking antibody nimotuzumab. Considering the majority of GB patients with unmethylated MGMT promoter status, activation of Akt or mTORC1 signaling was associated with a benefit from nimotuzumab treatment. A clear trend towards a benefit from nimotuzumab therapy was also detectable in the whole study cohort using activation of mTORC1 as a marker for dichotomy. We believe that our results constitute a basis for further investigation of nimotuzumab or other EGFR- and mTOR-inhibitors in selected patient cohorts using the reported criteria as candidate predictive biomarkers.

## Additional files

Additional file 1: **Figure S1.** Representative images of histological subclassifications. Representative images of immunohistochemical staining for HIF-1α, P-PRAS40, P-RPS6 and Iba1 from FFPE tumor specimens of below and equal to (low) and above (high) median marker frequency. (TIF 13094 kb)

Additional file 2: **Figure S2.** Correlation of perinecrotic HIF-1α and necrosis. Correlation of perinecrotic HIF-1α and necrosis in a bivariate plot with a linear regression analysis. P and r^2^ values as indicated. (TIF 64 kb)

Additional file 3: **Figure S3.** Distribution of histology markers in treatment arms. A, one way analysis with outlier box plot of necrosis, HIF-1α in perinecrotic or in vital central tumor regions, P-RPS6, P-PRAS40 and Iba1 in tumors of patients treated with nimotuzumab (nimo) or placebo (cont). B, one way analysis with outlier box plot of P-RPS6 and P-PRAS40 in tumors with methylated or unmethylated MGMT promoter. *P*-value calculated using Student’s *t*-test. (TIF 495 kb)

Additional file 4: **Figure S4.** Survival analyses and schemes of signal transduction. A, Weibull parametric analysis of P-PRAS40 and time to progression in patients treated with nimotuzumab (left panel) or placebo (control, right panel). B, scheme of a nutrient sensing via mTORC1 and effects on cellular adaptation and necrosis. Cells with an intact mTORC1 sensor inhibit mTORC1 signaling during nutrient deprivation and hypoxia, despite signaling from EGFR preventing widespread necrosis (left panel). In contrast cells with a defective mTORC1 sensor fail to adequately inhibit mTORC1 in response to nutrient deprivation or hypoxia resulting in more widespread areas of necrosis (right panel). C, scheme of mTORC1 signal transduction to S6 kinase 1 (S6 K1). S6 K1 phosphorylates both RPS6 at Ser 240/244 as well as mTOR at Ser 2448. D, survival analysis depending on Iba1 staining frequency (median split, above median: high, below and equal to median low). *P* values were calculated using the Wilcoxon test. (TIF 559 kb)
