# Supplementary figures and images for: Akt and mTORC1 signaling as predictive biomarkers for the EGFR antibody nimotuzumab in glioblastoma
Source: Acta Neuropathol Commun. 2018 Aug 21;6:81. doi: 10.1186/s40478-018-0583-4 (PMC6102828; doi:10.1186/s40478-018-0583-4)

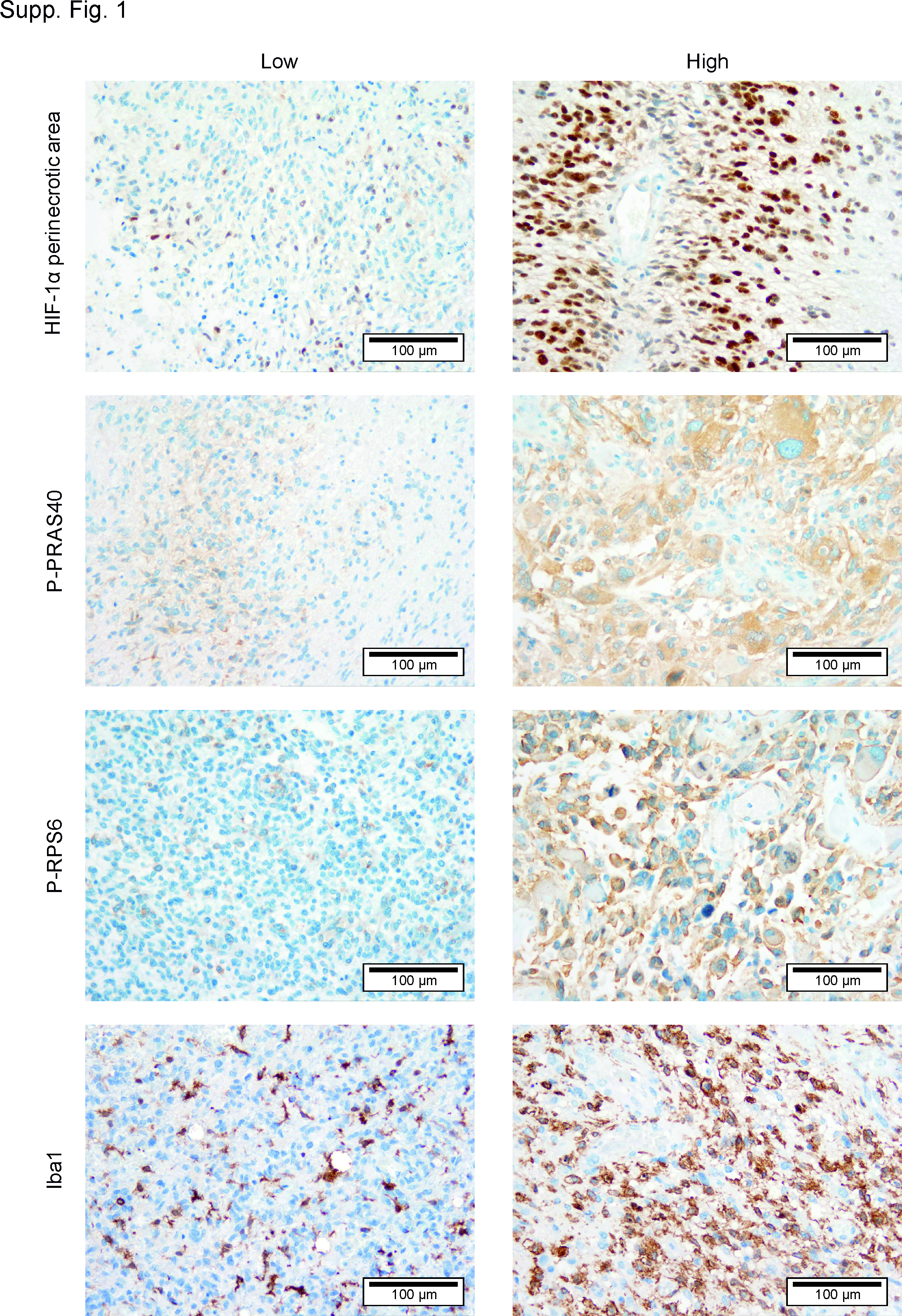

Supplement: Supplementary file 1 — Figure S1. Representative images of histological subclassifications. Representative images of immunohistochemical staining for HIF-1α, P-PRAS40, P-RPS6 and Iba1 from FFPE tumor specimens of below and equal to (low) and above (high) median marker frequency. (TIF 13094 kb) [file 40478_2018_583_MOESM1_ESM.tif]

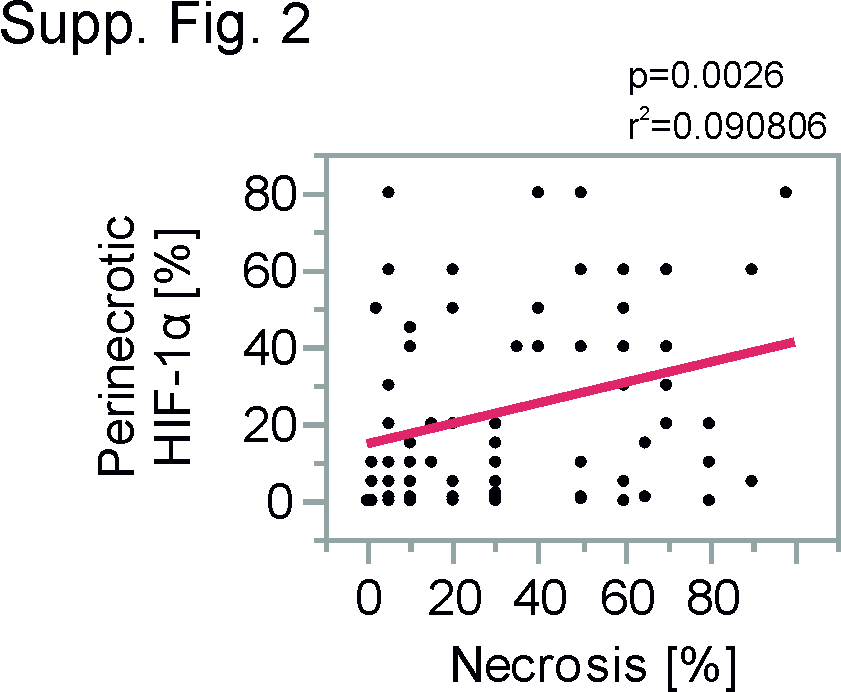

Supplement: Supplementary file 2 — Figure S2. Correlation of perinecrotic HIF-1α and necrosis. Correlation of perinecrotic HIF-1α and necrosis in a bivariate plot with a linear regression analysis. P and r2 values as indicated. (TIF 64 kb) [file 40478_2018_583_MOESM2_ESM.tif]

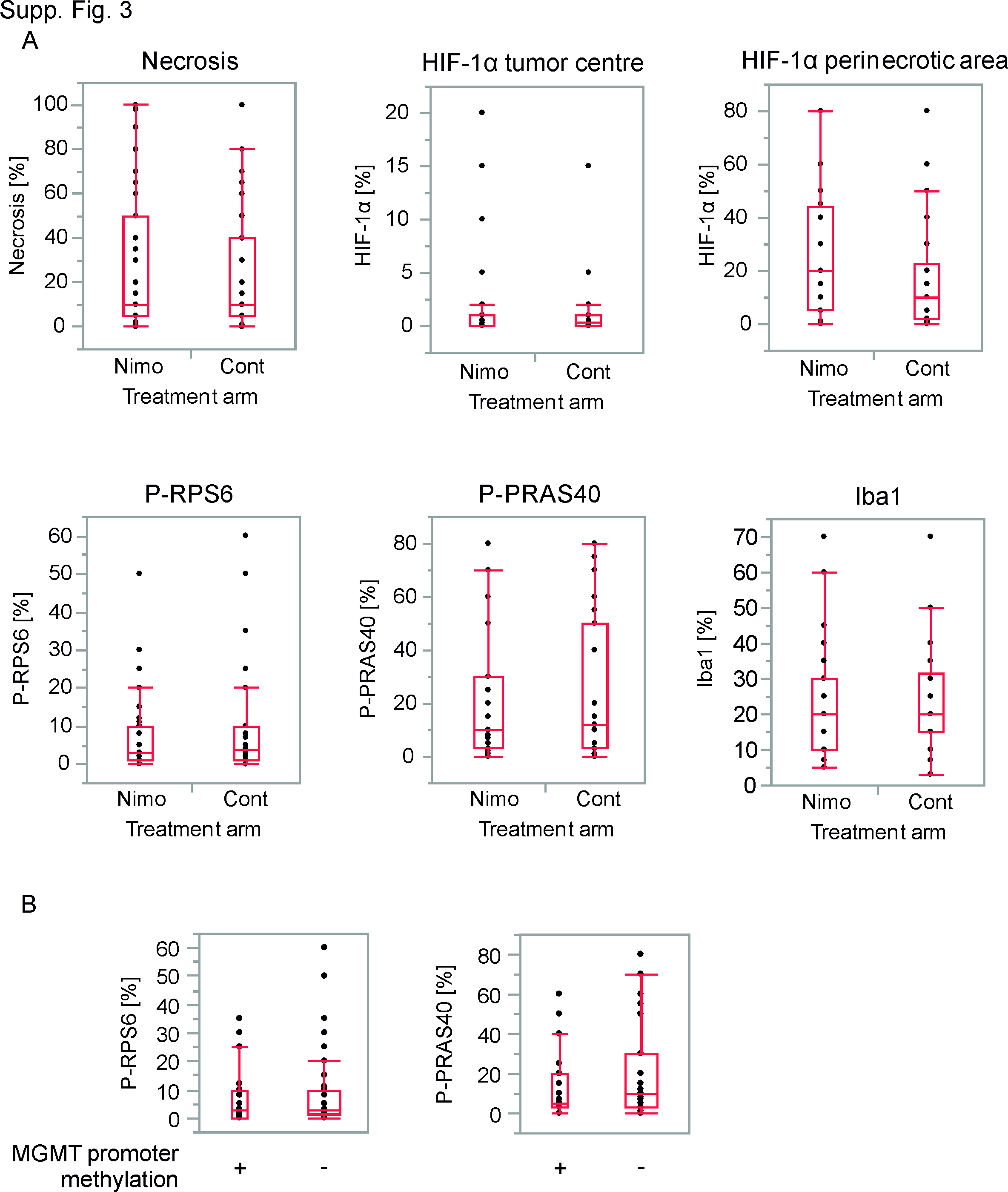

Supplement: Supplementary file 3 — Figure S3. Distribution of histology markers in treatment arms. A, one way analysis with outlier box plot of necrosis, HIF-1α in perinecrotic or in vital central tumor regions, P-RPS6, P-PRAS40 and Iba1 in tumors of patients treated with nimotuzumab (nimo) or placebo (cont). B, one way analysis with outlier box plot of P-RPS6 and P-PRAS40 in tumors with methylated or unmethylated MGMT promoter. P-value calculated using Student’s t-test. (TIF 495 kb) [file 40478_2018_583_MOESM3_ESM.tif]

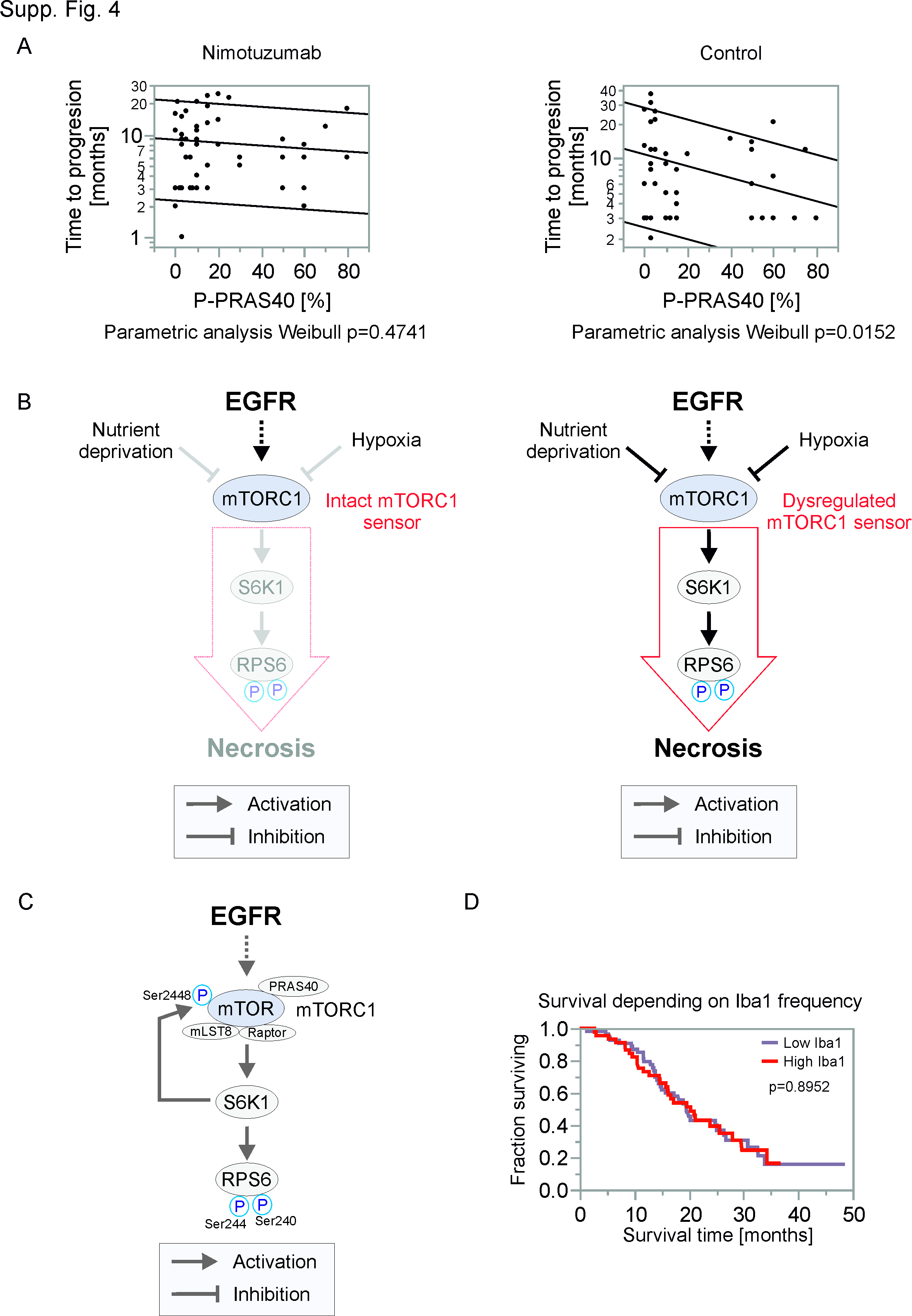

Supplement: Supplementary file 4 — Figure S4. Survival analyses and schemes of signal transduction. A, Weibull parametric analysis of P-PRAS40 and time to progression in patients treated with nimotuzumab (left panel) or placebo (control, right panel). B, scheme of a nutrient sensing via mTORC1 and effects on cellular adaptation and necrosis. Cells with an intact mTORC1 sensor inhibit mTORC1 signaling during nutrient deprivation and hypoxia, despite signaling from EGFR preventing widespread necrosis (left panel). In contrast cells with a defective mTORC1 sensor fail to adequately inhibit mTORC1 in response to nutrient deprivation or hypoxia resulting in more widespread areas of necrosis (right panel). C, scheme of mTORC1 signal transduction to S6 kinase 1 (S6 K1). S6 K1 phosphorylates both RPS6 at Ser 240/244 as well as mTOR at Ser 2448. D, survival analysis depending on Iba1 staining frequency (median split, above median: high, below and equal to median low). P values were calculated using the Wilcoxon test. (TIF 559 kb) [file 40478_2018_583_MOESM4_ESM.tif]
